# ﻿A new species of *Prunus* subgen. *Cerasus* from Central China

**DOI:** 10.3897/phytokeys.199.84354

**Published:** 2022-06-02

**Authors:** Song-Zhi Xu, Qi-Liang Gan, Zhen-Yu Li

**Affiliations:** 1 School of Life Science, Nantong University, Nantong, Jiangsu 226019, China Nantong University Nantong China; 2 Zhuxi Qiliang Biological Institute, Zhuxi, Hubei 442300, China Zhuxi Qiliang Biological Institute Zhuxi China; 3 State Key Laboratory of Systematic and Evolutionary Botany, Institute of Botany, Chinese Academy of Sciences, Beijing 100093, China Institute of Botany, Chinese Academy of Sciences Beijing China

**Keywords:** Central China, flowering period, *Prunuswangii*, wild cherry

## Abstract

A new species, *Prunuswangii* Q.L.Gan, Z.Y.Li & S.Z.Xu from western Hubei, Central China is described and illustrated. It is morphologically similar to *P.clarofolia* Schneid. and *P.pseudocerasus* Lindl., but differs in larger height, nearly erect branches, densely and horizontally arranged lenticels, straight lateral veins of leaves, persistent brownish bracts, reflexed and entire calyx lobes, 2-lobed petals with narrowly triangular sinus, earlier flowering and broadly ellipsoid fruits. Furthermore, *P.wangii* blooms in late February and the colour of flower changes with time, which makes it possible to be a new breeding material for ornamental cherry with early spring blooms.

## ﻿Introduction

There are extremely rich germplasm resources of wild *Prunus*, especially for P.subgen.Cerasus (Mill.) A. Gray, in Central China (Koehne 1912; Yu and Li 1986; Li and Bartholomew 2003). In recent years, field investigations on 15 species of P.subgen.Cerasus were carried out by Qi-Liang Gan in western Hubei Province including Zhuxi, Zhushan, Badong, Shennongjia, Nanzhang, Danjiangkou and so on. Wild populations of *P.canescens* Bois and some other species which were not recorded by Flora of China (Li and Bartholomew 2003) and the revised checklist (Xia and Tong 2018) have been rediscovered during the fieldwork. In 2021, Gan found an unknown species of wild cherry in Zhangjiashan Village and Tianbaozhaichachang, Zhuxi County. Based on field surveys, morphological and phenological studies and taxonomic literature research, we concluded that this species should be included in P.subgen.Cerasus (Mill.) A. Gray (Shi et al. 2013), but, as it differed from previously described taxa, we named and described it as a new species herein.

## ﻿Materials and methods

Specimens of the putative new species were collected in Zhuxi County of Hubei Province in 2021. Comparisons with its relatives were made by consulting specimens stored in PE or some virtual specimen databases (HIB, CCAU, KUN, IBK, IBSC, CVH, JSTOR). All morphological characters were measured with dissecting microscopes and were described using the terminology presented by Harris and Harris (1994).

## ﻿Taxonomic treatment

### ﻿ Prunus wangii

Taxon classificationPlantaeRosalesRosaceae

Q.L.Gan, Z.Y.Li & S.Z.Xu sp. nov.

5A88B299-8B18-568A-93D3-77EF99D13FB3

urn:lsid:ipni.org:names:77299024-1

Fig. 1

#### Diagnosis.

*Prunuswangii* Q.L.Gan, Z.Y.Li & S.Z.Xu is similar to *P.clarofolia* Schneid. and *P.pseudocerasus* Lindl., but the new species can be easily distinguished from the latter two species by its larger trees, densely and horizontally arranged lenticels, straight lateral veins of leaves, persistent brownish bracts, 2-lobed petals with narrowly triangular sinus and broadly ellipsoid fruits.

**Figure 1.**
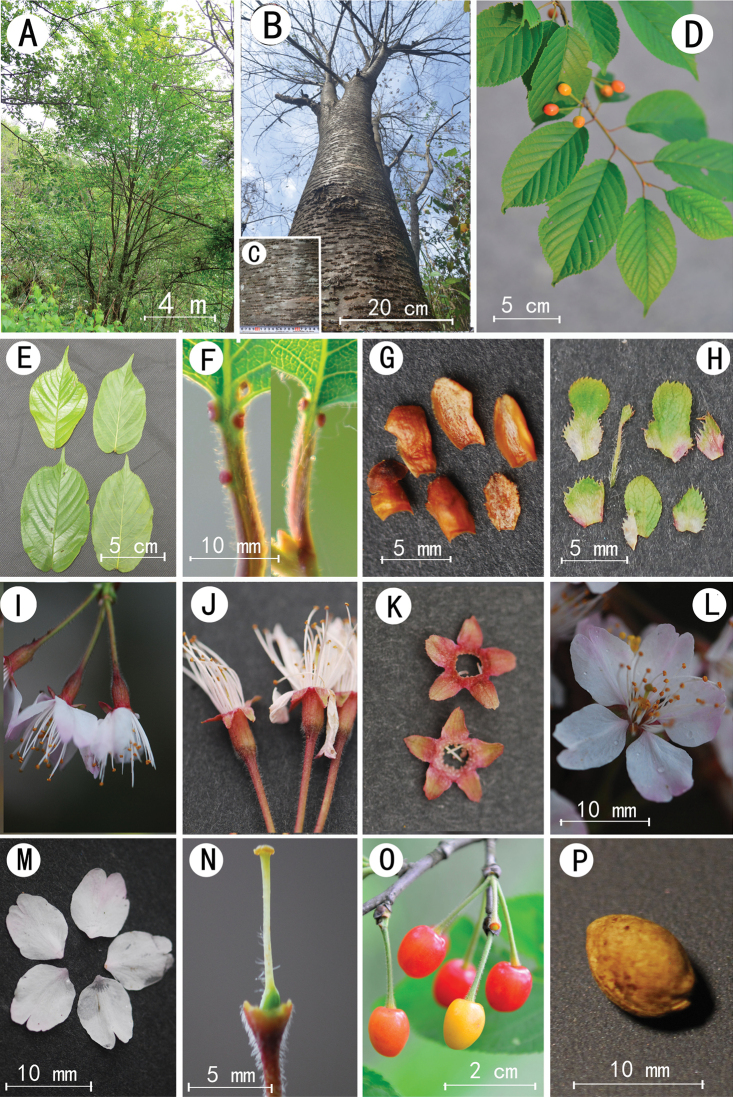
*Prunuswangii* sp. nov. **A** crown **B** trunk **C** bark **D** fruiting branches **E** leaf blades **F** petioles and glands **G** internal bud scales **H** involucres and bracts **I** inflorescence **J** pedicels and hypanthia **K** calyces **L** corolla **M** petals **N** ovary and style **O** fruits **P** seed.

#### Type.

China, Hubei Province, Zhuxi County, Quanxi Town, Zhangjiashan Village, in mixed forest on hillside, alt. 700 m, 26 February 2021, Q.L. Gan 3237-1 (holotype: PE!; isotype: PE!)

#### Description.

Trees, deciduous, to 21 m tall (flowering well even when less than 6 m tall), DBH to 30 cm. Bark dark grey, lenticels distinct, transversely elliptic, densely arranged in horizontal lines; crown ovoid. Branchlets grey or taupe, sparsely pilose when young, later glabrate. Winter buds ovoid, ca. 2.5 mm long, internal bud scales brown, glabrous outside, densely pilose inside. Stipules linear, 6–8 mm long, with laciniate or lacerate and gland-tipped lobes, caducous. Leaves alternate, brownish-green when young; petiole 8–12 mm long, sparsely pubescent, lavender brown adaxially, longitudinally grooved; leaf blade elliptic or obovate, 5–11 cm long, 3–6.4 cm wide, base shallowly cordate, rounded or sometimes obliquely cuneate, base of blade or apical part of petiole with or sometimes without 1–3 purple discoid glands, margin biserrate and uniserrate, serrations acute, with a small apical gland, apex cuspidate or acuminate; adaxial surface deep green, glabrous, abaxial surface pale green, white pubescent along veins, margin ciliate; lateral veins 6–9 pairs, straight, veins slightly impressed on adaxially surface and raised abaxially. Inflorescences corymbose 4–6-flowered or umbellate 2–3-flowered; involucre green, orbicular-ovate, 2–5 mm long, 2–4 mm wide, abaxially glabrous, adaxially densely pilose; peduncle 2–11 mm long, subglabrous or sparsely white pubescent; bracts green, brownish when dry, fan-shaped, rarely linear, 2–7 mm long, 0.5–3.5 mm wide, proximal one larger, margin glandular-serrate, persistent in fruit. Flowers and leaves opening at same time (coetaneous) or nearly so. Pedicel 0.9–1.2 cm long, to 1.3–2 cm long in fruit, straight, thickened at apex, densely white pilose. Hypanthium campanulate, 5–6 mm long, 2.5–3 mm in diam., pilose outside; calyx lobes triangular, 2.5–3 mm long, margin entire, apex rounded or subacute, glabrous or pilose outside, at first patent, reflexed when flower fully expanded. Petals pink during early blooming, then white or slight pinkish tinged, broadly ovate, 7–11 mm long, 5–9 mm wide, apex 2-lobed, sinus narrowly triangular. Stamens 34–38, as long as or shorter than petals, glabrous, 6–11 mm long; filaments white; anthers dark yellow, broadly ellipsoid, 0.65–0.75 mm long. Ovary green, glabrous; style shorter than stamens, sparsely patent white-pilose below middle. Drupe broadly ellipsoid, 10–12 mm long, 8–10 mm in diam., glabrous, red at maturity, shiny, tasting sweet and sour. Stone flattened ovoid, 8–8.5 mm long, 5–5.5 mm wide, ca. 4 mm thick, fawn, surface almost smooth, ribbed on one side.

#### Phenology.

Flowering from late February to late March, flowers and leaves opening at same time (coetaneous) or nearly so; fruiting from April to May.

#### Distribution and habitat.

*Prunuswangii* is known from only two populations composed of ca. 20 individuals in Zhangjiashan Village, Quanxi Town and Tianbaozhaichachang, Tianbao Town, Zhuxi County. It grows sparsely in mixed forests on hillsides at elevations from 600 to 800 m. The main companion species include trees: *Populusadenopoda* Maxim., *Liquidambarformosana* Hance, *Castaneaseguinii* Dode, *Platycaryastrobilacea* Sieb. & Zucc., *Pinusmassoniana* Lamb., *Cunninghamialanceolata* (Lamb.) Hook., *Prunusconradinae* Koehne and Prunussubhirtellavar.ascendens (Maxim.) E.H.Wilson; shrubs: *Coriarianepalensis* Wall., *Rubuslambertianus* Ser., *Rubuscoreanus* Miq., *Linderaglauca* (Sieb. & Zucc.) Bl. and Rosabanksiaevar.normalis Regel; vines: Hederanepalensisvar.sinensis (Tobl.) Rehd., *Lonicerajaponica* Thunb., *Clematisflorida* Thunb. and *Puerariamontana* (Lour.) Merr.; herbs: *Chrysanthemumindicum* L., *Miscanthusfloridulus* (Lab.) Warb. ex Schum & Laut. and *Senecioscandens* Buch.-Ham. ex D. Don; ferns: *Cyrtomiumfortunei* J. Sm., *Athyriumfilix-femina* (L.) Roth, *Aspleniumvarians* Wall. ex Hook. & Grev. and Pteriscreticavar.nervosa (Thunb.) Ching & S. H. Wu.

#### Etymology.

The species is named in honour of Professor Wen-Tsai Wang (1926–), a taxonomist at the Institute of Botany, the Chinese Academy of Sciences, who has devoted over 60 years to taxonomic studies of Ranunculaceae, Gesneriaceae, Boraginaceae, Urticaceae and many other families and the floristic research in eastern Asia.

#### Vernacular name.

Wen Cai Ying Tao (Chinese).

#### Conservation assessment.

We found *Prunuswangii* only in the towns of Quanxi and Tianbao, Hubei Province and estimated the population size to be ca. 20 individuals. The provisional conservation status is Critically Endangered (CR), based on criterion D (number of mature individuals fewer than 50) (IUCN 2022).

#### Economic uses.

There are more than 140 species of flowering cherries widely distributed in the Northern Hemisphere, but species blossoming in February are rare. Two species, *P.pseudocerasus* Lindl. and *P.campanulata* Maxim., have been used as important breeding materials of flowering cherries with early spring blooms since their introduction into Japan (Ohwi and Ohta 1973; Ohba 2001; Wang 2014). *Prunuswangii* will provide a new breeding material for early flowering ornamental cherries. The timber of this species is hard and was used to make chopping blocks and furniture by the local people. The mature fruit is red, sweet or slightly bitter and is eaten by the local people.

#### Paratypes.

China. Hubei: Zhuxi County, Quanxi Town, Zhangjiashan Village, in mixed forest on hillside, alt. ca. 700 m, 11 March 2021, Qi-Liang Gan 3238 (PE!); the same locality, 21 April 2021, Qi-Liang Gan 3239-1, 3239-2 and 3239-3 (PE!); Zhuxi County, Tianbao Town, Tianbaozhaichachang, in mixed forest on hillside, alt. ca. 750 m, 11 March 2021, Qi-Liang Gan 3240 (PE!).

## ﻿Results

*Prunuswangii* is morphologically similar to *P.clarofolia* and wild *P.pseudocerasus*. These three species bear red, sweet fruits known as ‘yeyingtao’ meaning wild fruit cherries. The diagnostic features of these three species are listed in Table 1.

**Table 1.** Morphological comparisons of *Prunusclarofolia*, *P.pseudocerasus* and *P.wangii*.

Characters	*P.clarofolia*	*P.pseudocerasus*	*P.wangii*
Habit	shrubs or smaller trees, 2.5–8 (–13)^*^ m tall	small trees, rarely shrubs, (2–) 4–8 m tall	trees, up to 21 m tall
Bark	dark grey, lenticels usually sparse	grey-brown, lenticels usually discontinuously arranged in horizontal lines	dark grey, lenticels densely arranged in horizontal lines
Leaf blades	ovate, ovate-elliptic or obovate-elliptic	ovate, oval or oblong-ovate	elliptic or obovate
Lateral veins	7–12 pairs, curved	9–11 pairs, curved	6–9 pairs, straight
Flowering (in Zhuxi county)	early April to late May, flowers and leaves opening at same time (coetaneous)	mid-February to late March, flowers opening before leaves (precocious)	late February to late March, flowers and leaves opening at same time (coetaneous) or nearly so
Bracts	green, persistent in fruit	greenish, but brownish when dry, deciduous after anthesis	green, but brownish when dry, persistent in fruit
Hypanthium	campanulate, glabrous	campanulate to broadly campanulate (some cultivars), outside pilose	campanulate, outside pilose
Calyx lobes	reflexed, margin glandular serrate or entire	spreading or scarcely reflexed, entire	reflexed, entire
Petals	pink or white, 7–9 × 5–8 mm, entire, crenate or erose	pink or white, 8–13 × 3.5–9 mm, apex emarginate or 2-lobed, sinus triangular	pink during early blooming, then white to slightly pinkish tinge, 7–11 × 5–9 mm, apex 2-lobed, sinus narrowly triangular
Stamens	20–30, shorter to longer than the petals	30–50, shorter than the petals	34–38, as long as or shorter than the petals
Style	pilose below the middle	glabrous	pilose below the middle
Drupe	ellipsoid, 7–9 × 4–5 mm, red, sweet and sour taste	globose, 9–13 mm in diam., red, rarely yellow or white (cultivars), sweet or sweet and sour taste	broadly ellipsoid, 10–12 × 8–9.5 mm, red, sweet and slightly bitter taste

*Yu & Li (1986, p.54) recorded *P.clarofolia* as ‘shrubs or small trees, 2.5–20 m tall’. ‘20 m’ is incongruent with ‘small trees’ and we think it should be 12 m. Furthermore, based on specimen records and fieldwork, this species is up to 13 m tall.

*Prunuswangii* resembles *P.clarofolia* in the dark grey old bark, green bracts, pilose style, campanulate hypanthium, reflexed calyx lobes, pink or white petals and flowers and leaves opening at the same time. *Prunuswangii* differs from *P.clarofolia* in having lenticels densely arranged in horizontal lines (vs. lenticels usually sparse), straight (vs. curved) lateral veins, hypanthium outside pilose (vs. glabrous), petals 2-lobed at apex, sinus narrowly triangular (vs. entire, crenate or erose), stamens 34–38 (vs. 20–30), flowering from late February to late March (vs. from early April to late May).

*Prunuswangii* can be distinguished from *P.pseudocerasus* in height, up to 21 m tall (vs. less than 8 m), having dark grey (vs. greyish-brown) bark, lenticels densely arranged in horizontal lines (vs. discontinuously arranged in horizontal lines), straight (vs. curved) lateral veins, style pilose below the middle (vs. glabrous), broadly ellipsoid (vs. globose) drupe, flowers and leaves opening at same time (vs. flowers opening before leaves).

Besides, *P.wangii* is also somewhat similar to *P.dielsiana* C. K. Schneid., but the latter can be easily distinguished by the densely yellowish to yellowish-brown pilose twigs, 10–13 pairs of lateral leaf veins, bracts with long stalked glands, calyx lobes ca. 1.5–2 times as long as the hypanthium, flowering from March to April and flowers opening before leaves.

## Supplementary Material

XML Treatment for Prunus wangii
